# Management of tobacco use in residential addiction services: professional and patient perspectives

**DOI:** 10.1192/j.eurpsy.2026.10450

**Published:** 2026-07-31

**Authors:** P. Caravaca, F. Calvo

**Affiliations:** Departament de Pedagogia, Universitat de Girona, Girona, Spain

## Abstract

**Introduction:**

In residential resources for substance use disorders (SUD), tobacco use is widely tolerated despite its health impact and the evidence showing that cessation does not worsen SUD course or psychiatric symptoms.

**Objectives:**

To describe tobacco management in therapeutic communities and reintegration housing.

To quantify prevalence, dependence, and motivation to quit.

To identify gaps between evidence and clinical practice.

**Methods:**

Qualitative design with a descriptive quantitative component. Two questionnaires (professionals/patients) and focus groups were conducted in 2 therapeutic communities and 3 reintegration apartments (Catalonia, Sep–Nov 2024). Sample: 29 professionals and 53 patients. Instruments: sociodemographic variables, smoking status, Fagerström Test for Nicotine Dependence, Adams & Biener’s (1992) Willingness to Quit Scale, and the Stages of Change Assessment (Becoña & Lorenzo, 2004).

**Results:**

Professionals (n=29): 69% had no training in tobacco management; 20.7% were smokers. All centers allowed smoking; 72.4% agreed with this policy. Among smoking staff, 48.3% reported smoking with patients; 62.1% preferred to postpone cessation.

Patients (n=53): 87.8% were daily smokers; 51.1% had high dependence; 65.1% were in precontemplation. While 71.5% agreed with allowing smoking, 58.1% perceived insufficient support to quit; only 11.6% had attempted cessation during treatment.

Qualitative themes: (a) tobacco as an emotional regulator and “substitute” for other drugs; (b) ambiguous professional modeling (smoking together); (c) discontinuity of “smoke-free” settings after inpatient detoxification; (d) belief that cessation increases relapse risk despite evidence to the contrary.

**Conclusions:**

A policy–practice gap persists: high prevalence and tacit validation of tobacco coexist in settings promoting integral health. Systematic integration of tobacco treatment (psychological and pharmacological), team training, and continuity of smoke-free environments could improve both SUD and mental health outcomes. Institutional policies must align with evidence and reconsider professional modeling.

**Disclosure of Interest:**

None Declared

